# Clinical Characteristics of Pediatric Coats' Disease With Retinal Cyst Using Wide-Angle Fluorescein Angiography

**DOI:** 10.3389/fmed.2021.709522

**Published:** 2021-10-27

**Authors:** Jing-Hua Liu, Guangda Deng, Jing Ma, Liang Li, Yuxin Fang, Songfeng Li, Hai Lu

**Affiliations:** Beijing Tongren Hospital, Capital Medical University, Beijing, China

**Keywords:** pediatric, Coats' disease, retinal cyst, wide-angle fluorescein angiography, clinical characteristics

## Abstract

**Purpose:** To assess the demographic and treatment features of pediatric patients of Coats' disease with retinal cyst using wide-angle FA.

**Design:** A retrospective, hospital based, cross-sectional study.

**Participants:** Pediatric patients of Coats' disease underwent wide-angle FA.

**Methods:** A retrospective review of pediatric patients of Coats' disease who underwent wide-angle FA at a single center from January 2015 to July 2020. Demographic and treatment features were compared between patients with or without retinal cyst.

**Main Outcome Measures:** Demographic and treatment outcomes.

**Results:** There were 123 pediatric Coats' patients in our study, and 18.70% (23/123) of the patients developed complications with retinal cyst, 73.9% (17/23) of the retinal cysts were located in the inferior-temporal quadrant and 82.6% (19/23) of the retinal cysts were located in the peripheral retina anterior to the vortex veins. Compared with patients without retinal cyst, patients with retinal cyst had more clock-hours of telangiectasia on FA (7.32 vs. 5.41, *p* = 0.031), and may need more total treatments (7.47 vs. 3.53, *p* = 0.023) including laser photocoagulation (4.08 vs. 2.31, *p* = 0.019) or intravitreal anti-VEGF (3.13 vs. 2.23, *p* = 0.039), and also required a longer time for telangiectasia resolution (22.33 vs. 18.53 months, *p* = 0.043).

**Conclusion:** Pediatric patients with Coats' disease complicated by retinal cyst presented with more clock-hours of telangiectasia on FA and needed more total treatments and longer time for telangiectasia resolution.

## Introduction

Coats' disease is a congenital, idiopathic retinal telangiectasia characterized by intraretinal and/or subretinal exudation leading to progressive exudative retinal detachment without retinal or vitreous traction (1, 2). It remains a great challenge of diagnosis and treatment because of its varied clinical presentation, and the majority of the cases present with advanced stages ending with poor visual acuity prognosis in spite of aggressive treatment (3, 4).

Retinal cyst is defined as a fluid-filled space derived from or in the retina (5), and may be related to focal anoxia or degeneration caused by long-standing retinal detachment such as Coats' disease (6). There are a few reports about Coats' disease complicated with retinal cyst, but these have either been single cases or have given few details of its clinical and treatment features (7, 8).

We hereby retrospectively reviewed a case series of pediatric Coats' disease and gave a descriptive analysis of pediatric Coats' disease complicated with retinal cyst, using RetCam III imaging combined with wide-angle fluorescence angiography (FA).

## Methods

The medical and imaging records of 123 children (≤ 18 years) with Coats' disease who underwent treatment with the surveillance of RetCam III imaging combined with wide-angle FA in Beijing Tongren Hospital from January 2015 to July 2020 were retrospectively reviewed. This study has been approved by the Hospital Board and was performed in accordance with the Declaration of Helsinki and we have obtained informed consent from guardians of all the patients. To be included in the study, the patients had to show idiopathic retinal telangiectasia defined as irregular, dilated small, or medium vessels manifested on wide-angle FA, and/or retinal lipid exudation. Patients with uncertain diagnosis and without complete records were excluded.

Coats' disease was staged based on a previously published classification system (9): Stage 1 (only retinal telangiectasia); Stage 2a (telangiectasia and extrafoveal exudation); Stage 2b (telangiectasia and foveal exudation); Stage 3a (subtotal exudative retinal detachment); Stage 3b (total exudative retinal detachment); Stage 4 (total exudative retinal detachment and secondary glaucoma); Stage 5 (phthisis bulbi).

Fundus examination under anesthesia including indirect ophthalmoscopy, color photography and FA with Retcam III was arranged for both eyes of all the patients, each patient was given an intravenous bolus of 20% sodium fluorescein (0.1 ml/kg) before performing FA. Color Doppler ultrasonography and OCT were used to document the presence and extent of retinal detachment and retinal cyst in some cases.

Ablative therapies such as 532-nm laser, cryotherapy, or both were administrated to the areas of retinal non-perfusion and telangiectatic vessels according to FA findings. Cryotherapy was used only when there was confluent extensive exudation of the telangiectatic vascular areas. Intravitreal injection of Ranibizumab (Lucentis; Genentech Inc., South San Francisco, CA, USA) was used for patients with exudative retinal detachment (stage 3 or over). Vitreoretinal surgery, which included any combination of subretinal fluid drainage, pars plana vitrectomy were arranged for some cases.

The patients were monitored between 4 and 12 weeks after treatment, and further treatment was undertaken if there was a lack of telangiectatic vascular resolution or an increase of exudation at follow-up appointment. Demographic data including age at presentation, sex, laterality, clinical features such as pre- and postoperative visual acuity, Coats' disease stages, macular involvement (macular involvement was defined in this study as retinal detachment or yellow hard exudate involving the macular fovea and with a diameter more than one optic disc), retinal cyst and its location, clock hours of FA telangiectasia, treatment modalities, and anatomical prognosis were reviewed and compared between the two groups with or without retinal cysts. Snellen visual acuities were recorded and converted to logarithm of the minimum angle of resolution (log MAR) units for statistical evaluation.

Statistical analysis was performed using SPSS software (version 17.0, SPSS Inc., Chicago, IL, USA). Kolmogorov-Smirnov test was used to analyze the normal distribution of continuous variables, and Mann-Whitney U test was used to compare continuous variables if the data did not follow a normal distribution, Student sample *t*-test was used to compare continuous variables if the data followed a normal distribution between groups with or without retinal cyst; Fisher exact test was used to compare categorical variables between groups and *p*-value of 0.05 or less was considered statistically significant.

## Results

Demographic features of the study participants: 123 children with Coats' disease (123 eyes) were identified in this study, with at least 6 months follow-up. Overall average age at presentation was 5.59 years (5.59 ± 2.40) and the predominant sex was male (113/123, 91.87%). Retinal cyst was complicated in 23 patients (23 eyes, 23/123, 18.70%), and a comparison of demographic features between patients with and without retinal cyst were listed in Table 1. Statistical analysis revealed no significant difference in presenting age, sex, affected eye (Table 1), preoperative visual acuity, Coats' disease stages and macular involvement between the two groups, but FA showed more clock hours of telangiectasia in the group of patients with retinal cyst compared with cases without retinal cyst (7.32 vs. 5.41, *p* = 0.031), and there was a trend for eyes in patients with retinal cyst to demonstrate more advanced stages of disease (stage 3A to 5) than those in patients without retinal cyst (73.91 vs. 50.0%, *p* = 0.023) (Table 2).

**Table 1:** Demographics of Coats' disease with or without retinal cyst.

**Variable**	**Patients with retinal cyst**	**Patients without retinal cyst**	***p*** **-value**
No. of patients (eyes)	23 (23)	100 (100)	
Age at presentation(years)			0.057
Mean ± SD	6.35 ± 3.28	5.41 ± 2.12	
Range	2–15	3–15	
Follow-up (months)			0.731
Mean ± SD	29.61 ± 11.30	32.02 ± 15.07	
Range	9–60	6–65	
Sex, no. (%)			0.933
Male	21 (91.30%)	92 (92.0%)	
Female	2 (8.70%)	8 (8.0%)	
Eye laterality, no. (%)			0.789
Right eye	11 (47.83%)	46 (46.0%)	
Left eye	12 (52.17%)	54 (54.0%)	

**Table 2:** Baseline ocular characteristics of Coats' disease with or without retinal cyst.

**Variable**	**Patients with retinal cyst**	**Patients without retinal cyst**	***p-*** **value**
No. of patients (eyes)	23 (23)	100 (100)	
Best-corrected visual acuity (log MAR) no. (%)			0.062
≤ 0.7	2 (8.70%)	4 (4.0%)	
1.70–0.7	3 (13.04%)	21 (21.0%)	
≥ 1.70	15 (65.22%)	58 (58.0%)	
Uncooperative	3 (13.04%)	17 (17%)	
Coats' disease stage, no. (%)			0.157
1	0 (0)	0 (0)	
2a	3 (13.04%)	17 (17.0%)	
2b	3 (13.04%)	33 (33.0%)	
3a	16 (69.57%)	44 (44.0%)	
3b	1 (4.35%)	5 (5.0%)	
4	0	1 (1.0%)	
5	0	0	
Macular involvement			0.238
Yes	19 (82.6%)	81 (81.0%)	
No	4 (17.4%)	19 (19.0%)	
Combined stages of Coats', no. (%)			0.023^*^
Stages 1 to 2B	6 (26.09%)	50 (50%)	
Stages 3A to 5	17 (73.91%)	50 (50%)	
Fluorescein angiography telangiectasia clock hours			0.031^*^
Mean ± SD	7.32 ± 2.73	5.41 ± 3.02	
Range	4–12	1–12	

Retinal cysts complicated in those cases with Coats' disease manifested as round, clear-demarcated, protuberant cystic pathologies located in posterior or peripheral retina. Retinal cysts were mostly located in the inferior-temporal quadrant (17/23, 73.91%) and the superior-temple quadrant (5/23, 21.72%), only 1 eye with retinal cyst located in the superior-nasal quadrant, and no eye with retinal cyst located in the inferior-nasal quadrant was seen in our case series; 82.60% (19/23) of the retinal cysts located in the peripheral retina (anterior to the vortex veins), and 17.39% (4/23) of the retinal cysts located in the posterior retina (posterior to the vortex veins).

**Figure 1:**
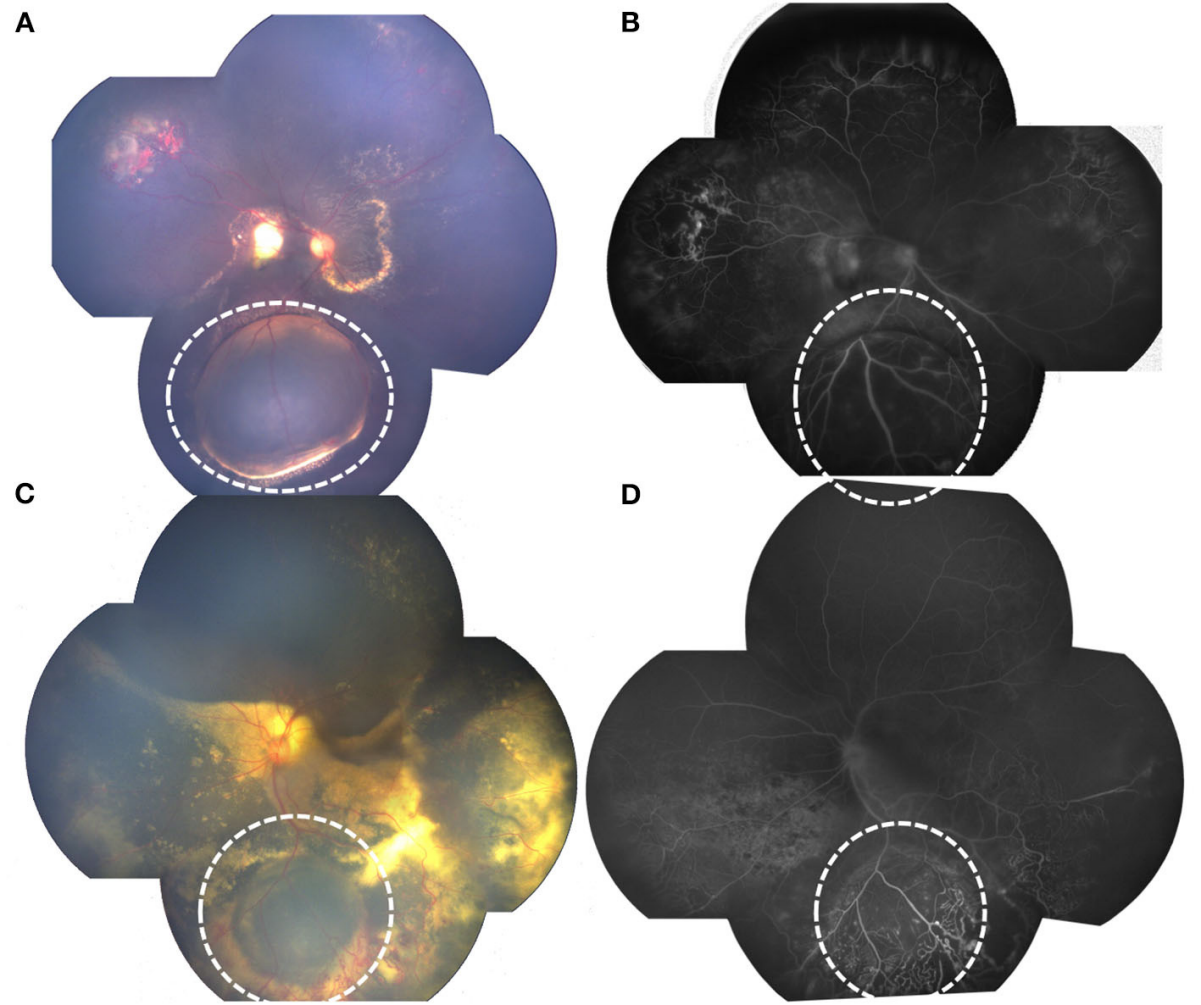
RetCam fundus and FA photographs showing Coats' disease with retinal cysts: **(A)** bullous retinal cyst in the inferior quadrant, surrounded with yellow hard exudation (white dotted circle); **(B)** FA of **(A)** showing well-demarked, protuberate pathology, with anterior layer capillary non-perfusion and late-phase punctate hyperfluorescence of the posterior layer, telangiectasia located at the edge of the cyst (white dotted circle). **(C)** retinal cyst (white dotted circle), surrounded with massive telangiectasia; **(D)** FA of **(C)** showing protuberate cyst, with anterior layer capillary non-perfusion and telangiectasia.

In addition to common clinical features on FA of Coats' disease such as widespread retinovascular telangiectasia, microaneurysms as well as capillary non-perfusion, retinal cyst on FA revealed round, well-demarcated, bullous pathologies with cystic cavity, with capillary non-perfusion areas in the anterior layer of the cyst and late-phase punctate hyperfluorescence of the posterior layer, also telangiectatic and aneurysmal vessels and multiple areas of peripheral capillary non-perfusion at or around the edge of the cyst (Figure 1).

**Figure 2:**
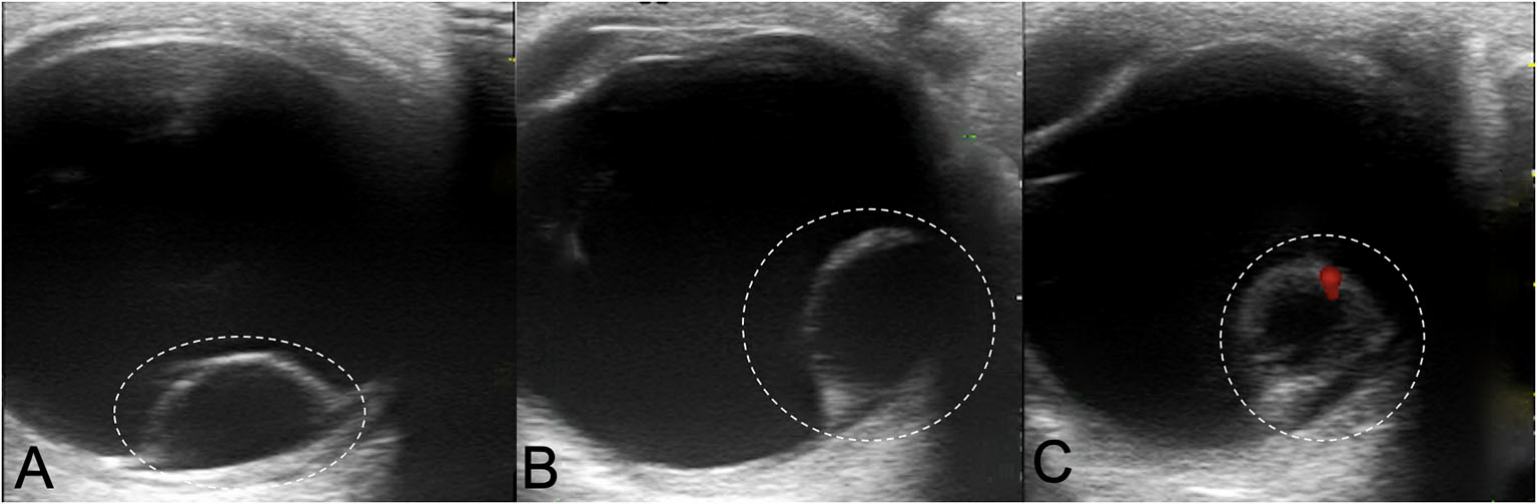
Color Doppler Image showing Coats' disease with retinal cysts: **(A,B)** intraocular cystic mass connected with the hyperechoic area of ocular wall (white dotted circle); **(C)** localized vascular signal on the anterior layer (red signal).

**Figure 3:**
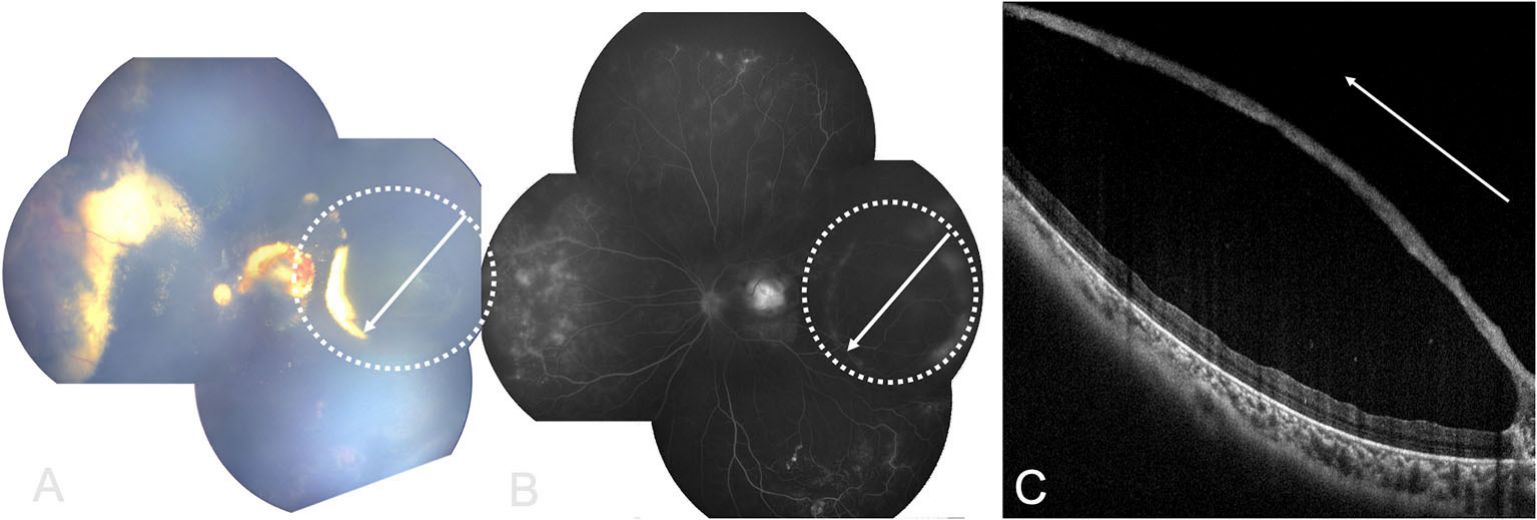
RetCam fundus, FA and OCT images showing Coats' disease with retinal cysts: **(A)** retinal cyst (white dotted circle), white arrow showing OCT scan direction; **(B)** FA of **(A)**, white arrow showing OCT scan direction; **(C)** OCT image showing retinal cyst with separation of the inner and outer layers of retina (white arrow showing OCT scan direction).

Color Doppler Image (CDI) showed intraocular cystic mass connected with the hyperechoic area of ocular wall (Figure 2), with localized vascular signal on the anterior layer (Figure 2C). OCT showed retinal cyst as separation of the inner and the outer layer of the retina (Figure 3).

Treatment features of patients in our study are listed in Table 3 and a comparison of treatment features between the two groups revealed that the group of patients with retinal cyst had greater total number of treatments (7.47 vs. 3.53, *p* = 0.023); and more use of laser photocoagulation (4.08 vs. 2.31, *p* = 0.019), and intravitreal anti-VEGF (3.13 vs. 2.23, *p* = 0.039).

**Table 3:** Treatment features of Coats' disease with or without retinal cyst.

**Treatment features**	**Patients with retinal cyst**	**Patients without retinal cyst**	***p-*** **value**
No. of patients (eyes)	23 (23)	100 (100)	
Number of total treatments per patient			0.023^*^
Mean ± SD	7.47 ± 3.01	3.53 ± 2.31	
Range	(4–12)	(1–12)	
Argon laser photocoagulation			0.019^*^
Number of treatments per patient			
Mean ± SD	4.08 ± 1.35	2.31 ± 0.99	
Range	(2–6)	(1–5)	
Anti-VEGF			0.039^*^
Number of treatments per patient			
Mean ± SD	3.13 ± 1.03	2.23 ± 1.57	
Range	(2–5)	(0–4)	

* *Statistically significant*.

Other treatment modalities such as cryotherapy, pars plana vitrectomy and subretinal fluid drainage were also used for these patients, but without statistically significant differences between the two groups (*p* = 0.072): 11 eyes of the 123 eyes (11/123, 8.9%) underwent cryotherapy, of which 3 eyes presented with retinal cyst and the other 8 eyes without retinal cyst; 8 eyes of the 123 eyes (8/123, 6.5%) underwent pars plana vitrectomy, of which 2 eyes presented with retinal cyst and the other 6 eyes without retinal cyst; 8 eyes of the 123 eyes (8/123, 6.5%) underwent subretinal fluid drainage, of which 3 eyes presented with retinal cyst and the other 5 eyes without retinal cyst.

**Figure 4:**
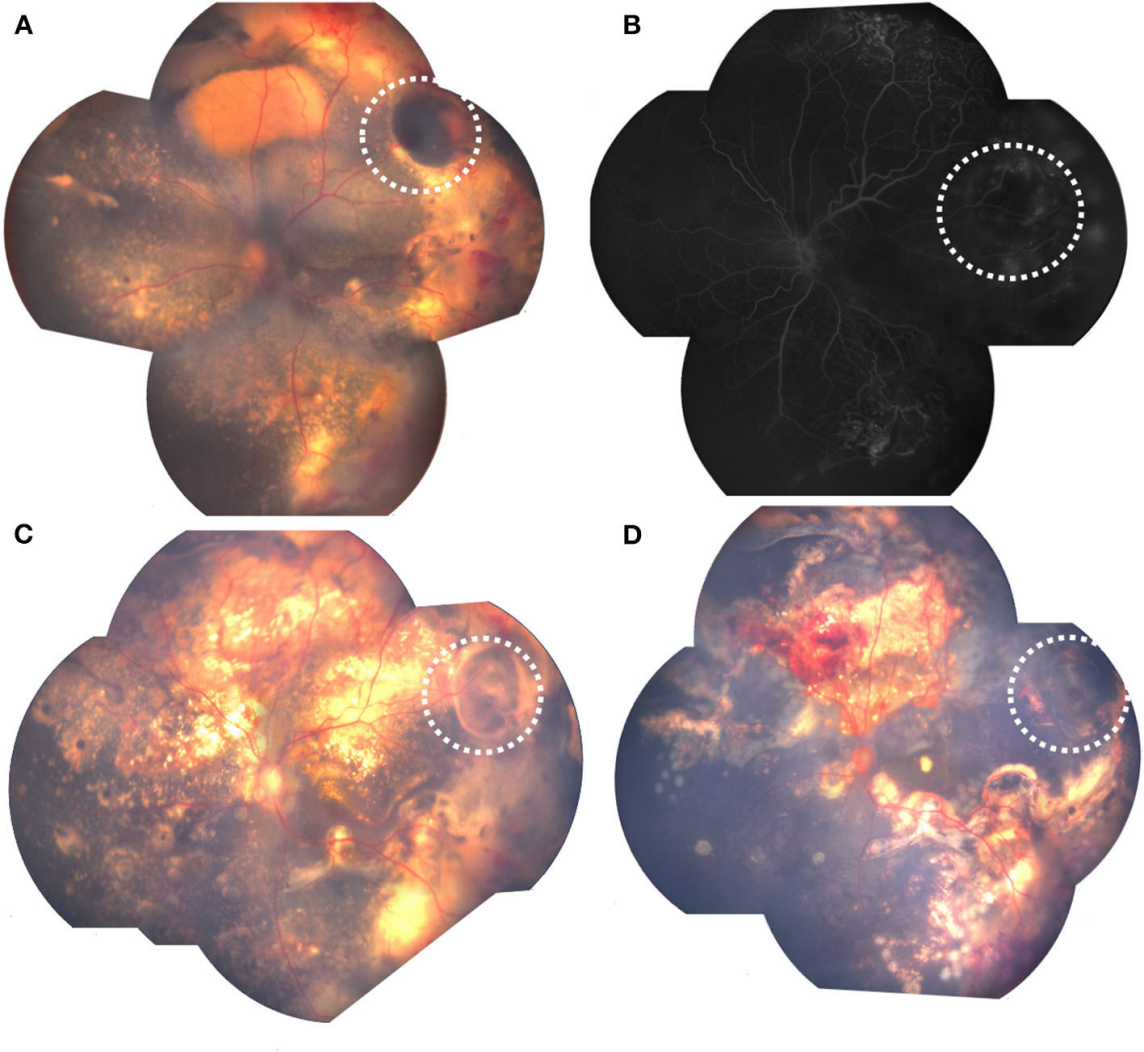
Pre-and postoperative fundus photographs of Coats' with retinal cyst.: **(A)** preoperative retinal cyst (white dotted circle), surrounded with exudative retinal detachment; **(B)** FA showing protuberate cyst (white dotted circle); **(C)** decreased retinal cyst (white dotted circle), with retinal attachment after 2 laser photocoagulation combined with intravitreal anti-VEGF; **(D)** retinal cyst resolution (white dotted circle), with retinal attachment after 2 additional laser photocoagulation.

**Figure 5:**
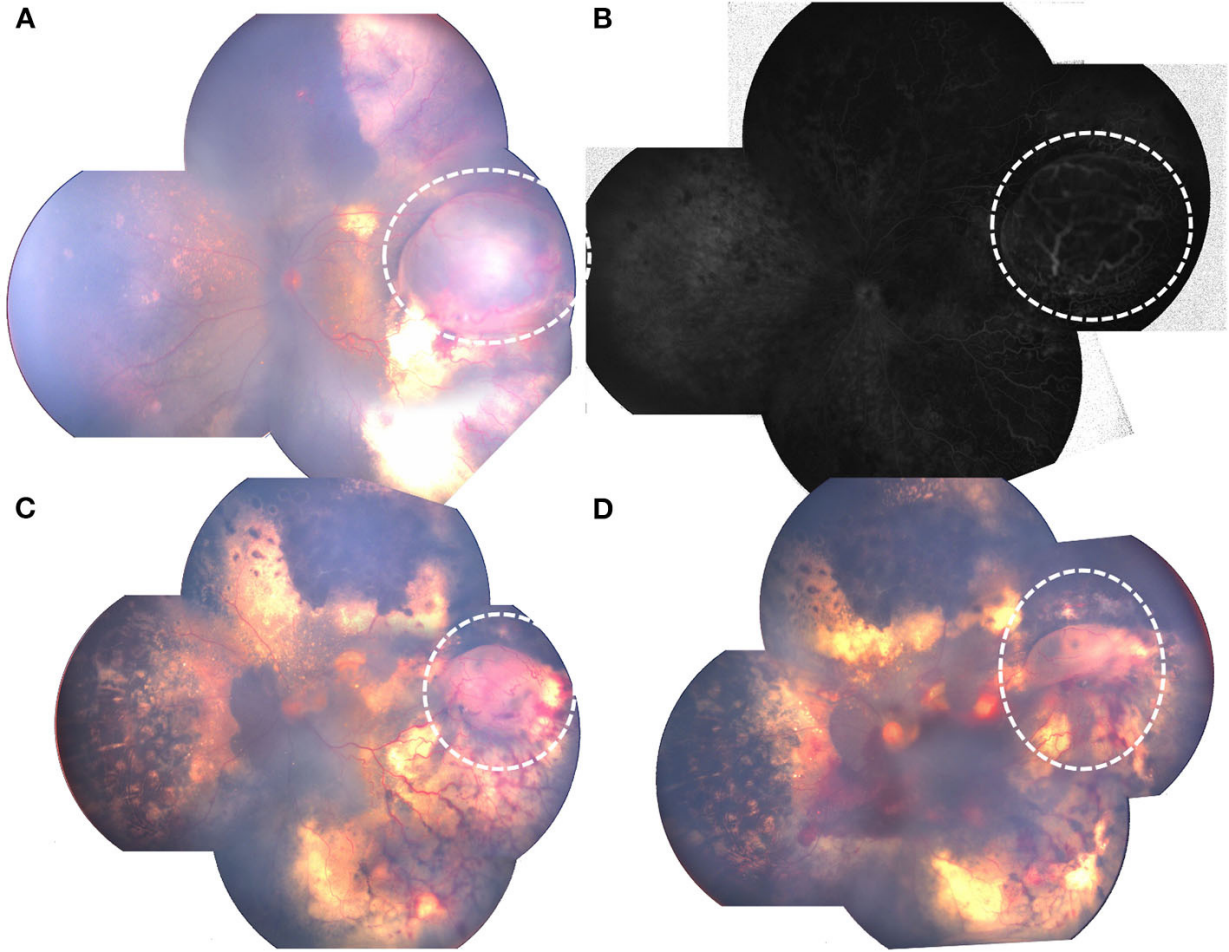
Pre-and postoperative fundus photograph of Coats' with retinal cyst.: **(A)** preoperative retinal cyst (white dotted circle); **(B)** FA showing protuberate cyst (white dotted circle); **(C)** decreased retinal cyst (white dotted circle) after 3 laser photocoagulation combined with intravitreal anti-VEGF; **(D)** partial retinal cyst resolution (white dotted circle), with minimal vitreous hemorrhage after 3 additional laser photocoagulation combined with 2 intravitreal anti-VEGF.

Figures 4, 5 showed fundus photographs of pre-and postoperative fundus photographs of patients with retinal cysts, indicating different degrees of retinal cyst resolution after combined treatment modalities.

Postoperative last-visit visual acuity and percentages of resolution of leaking telangiectasia were also compared and without statistically significant differences between the two groups, but patients with retinal cysts needed longer times for the resolution of leaking telangiectasia (22.33 vs. 18.53 months, *p* = 0.043) (Table 4).

**Table 4:** Outcomes of Coats' disease with or without retinal cyst.

**Outcomes**	**Patients with retinal cyst**	**Patients without retinal cyst**	***p-*** **value**
No. of patients (eyes)	23 (23)	100 (100)	
Best corrected visual acuity (log MAR) no. (%)			0.639
≤ 0.7	2 (2/23, 8.70%)	4 (4/100, 4.00%)	
1.70–0.7	4 (4/23, 17.39%)	23 (23/100, 23.00%)	
≥ 1.70	16 (16/23, 69.57%)	62 (62/100, 62.00%)	
Uncooperative	1 (1/23, 4.35%)	11 (11/100, 11.00%)	
Leaking telangiectasia resolution no. (%)			0.750
Resolved	18 (18/23, 78.26%)	87 (87/100, 87.00%)	
Not resolved	5 (5/23, 21.74%)	13 (13/100, 13.00%)	
Time to resolution (months)			0.043^*^
Mean ± SD	22.33 ± 7.98	18.53 ± 5.47	
Range	(15–33)	(9–29)	

## Discussion

Coats' disease represents a broad clinical spectrum of retinal vasculopathy and presents numerous challenges in the management across the disease spectrum (10, 11). Retinal cyst is usually complicated in long-standing rhegmatogenous or exudative retinal detachment such as Coats' disease, which causes focal retinal anoxia or focal retinal cell liquefaction and degeneration.

With the advent of wide-field portable RetCam III imaging, intraoperative FA can be obtained and thus facilitates full identification of abnormal retinovascular areas, where treatment such as laser or cryotherapy could be administrated under general anesthesia for pediatric patients (12–15). The purpose of the study was to investigate the clinical and treatment features of Coats' disease with retinal cyst compared with that without retinal cyst in pediatric patients underwent RetCam III wide-angle FA guided treatment.

We reviewed our clinical experience with Coats' disease in pediatric patients and found that several factors have not changed compared with previous studies (16–19), such as median age at presentation, predominant sex as male (113/123, 91.8%), predominant Coats' disease stage as stage 3a (48.78 vs. 42%). Our study also showed that patients with retinal cyst had more clock hours of telangiectasia on FA compared with patients without retinal cyst, indicating that patients with retinal cyst may had longer and much severe exudative retinal detachment that induced longer time of retinal anoxia or degeneration, which is the pathophysiological basis of retinal cyst formation.

We reviewed the literature and found only Vineet Mutha described the location of retinal cyst in a 5-year-old boy with Coats' disease staged as 3A, and the complicated retinal cyst located in the superonasal quadrant of the fundus (7). In our study, retinal cysts were mostly located in the inferior-temple quadrant and the superior-temporal quadrant, and 82.6% (19/23) of the retinal cysts located in the peripheral retina. We also found in our study that severe combined stages of Coats' disease (3A-5) had larger proportions of retinal cysts formation (*p* = 0.023). It is not difficult to interpret those results because subretinal fluid accumulated in the inferior quadrant because of gravity, or around the telangiectasia area, which is always located in the peripheral fundus, especially in cases with much severe stages, so long-time exudative retinal detachment may be located in the inferior and peripheral retina in cases with severe Coats' stages, thus forming the pathological basis of retinal cyst formation.

In addition to FA documentation of telangiectasia, light bulb aneurysm, peripheral non-perfusion, and cystoid macular edema, which are frequently seen in Coats' patients (20–22), patients with retinal cysts also demonstrated with well-demarcated, bullous cystic pathologies with the hypo-fluorescence anterior layer “flapping” in the vitreous cavity, late-phase hyper-fluorescence or capillary fluorescence leakage of the posterior layer of the cyst could also be seen, which is different from FA appearance of retinal detachment. Another differential diagnosis of FA appearance of Coats' disease with retinal cysts is retinoschisis, which is always bilaterally involved and without telangiectasia or light bulb aneurysm.

Ultrasonography has long been known to be an essential tool for diagnosis of ocular cysticercosis, and Coats' disease with retinal cyst could manifest as a cystic mass on ultrasonography mimicking a cysticercus cyst with scolex (10). Color Doppler image showed intraocular cystic masses with its hyperechogenic wall connected with the echo of ocular wall, also red vascular signals on the anterior layer, which can be differentiate from cysticercus scolex.

But a smaller retinal cyst (<2 diameters of optic disc) which was buried in extensive retinal detachment, was difficult to distinguish by FA or Ultrasonography.

Exudative retinal detachment complication in Coats' disease is difficult to differentiate from retinal cyst on a fundus photograph, but OCT may give an excellent differentiation as Figure 3 showed: retinal cyst manifested as separation of the inner and the outer layer of the neuroretina, while retinal detachment was the separation of neuroretina and the retinal epithelium.

Treatment of Coats' disease should be directed toward obliterating the telangiectasia by laser photocoagulation or cryotherapy (23–25), more advanced cases complicated by severe exudative retinal detachment may require combined therapies such as intravitreal anti-VEGF, or subretinal fluid drainage to facilitate resolution of the exudate or subretinal fluid (26, 27). For cases complicated with vitreoretinal traction or opacities, pars plana vitrectomy may be needed (28).

The average total number of treatments of all the patients in our study is 4.42, which is comparable with the latest studies in the 2010's (8, 15, 19). Our results also revealed that patients with retinal cyst had a greater total number of treatments, and more use of laser photocoagulation and intravitreal anti-VEGF, probably because of the increased number of hours of telangiectasia on FA of patients with retinal cyst which needs more treatments including laser photocoagulation and intravitreal anti-VEGF for the complete resolution of Coats' pathologies, furthermore, vascular telangiectasia may locate around the anterior edge of retinal cyst just as Figure 1 shows, and the protuberant cyst may make it much more difficult for the thorough laser photocoagulation of the telangiectasia area through binocular indirect ophthalmoscope. Comparisons of other treatments such as cryotherapy, pars plana vitrectomy or subretinal fluid drainage between the two groups in our study revealed no statistical differences, probably because a small number of patients underwent those treatments in our study, and future research with many more patients will be needed.

The presence of thick foveal exudation (stage 2B and above) usually portends a worse visual prognosis both preoperatively and postoperatively, our study showed that 70.27% (78/111) of the patients had postoperative visual acuity worse than log MAR 1.70, which was comparable to previous studies (29, 30), and statistical differences about postoperative visual acuity were not found between the two groups with or without retinal cyst, because most of the patients in our study presented with late Coats' disease stages accompanied by retinal detachment or thick foveal exudation involving the macular with no statistical differences in the macular involvement percentages (*p* = 0.238) between the two groups, hence the poor visual acuity prognosis.

Shields et al. reported that improvement of stabilization of the disease was achieved in almost 76% of their patients from an anatomic standpoint, and 85.37% (105/123) of the patients in our study had leaking telangiectasia resolution at last visit, with no statistical differences between the two groups (*p* = 0.750). However, our results showed that patients with retinal cysts needed longer time for resolution of leaking telangiectasia (22.33 vs. 18.53 months, *p* = 0.043), which may be explained by the fact that patients with retinal cysts had much more clock-hours of telangiectasia and needed longer time for total resolution of leaking telangiectasia.

This retrospective study has many limitations including its relatively short time of follow-up and the potential bias that patients in our study were mostly referred from other hospitals and had very severe pathologies with lower ages at presentation, so the retinal cyst percentage of 18.70% may not be suitable for all the Coats' patients. But it has its advantages of being the largest series of studies investigating the clinical characteristics of pediatric Coats' patients with retinal cyst. However, with the long life span of these patients, further studies with longer-term follow up and more paralleled cases is necessary to explore the visual and anatomical prognosis of these patients.

In summary, we have reviewed our experience with Coats' disease complicated with retinal cysts, and our results showed that 18.70% (23/123) of the Coats' patients may present with complications of retinal cyst, and retinal cysts were mostly located in the inferior-temporal quadrant and in the peripheral retina; patients with retinal cyst had more clock-hours of telangiectasia on FA, and may need more treatments including laser photocoagulation or intravitreal anti-VEGF, also longer time for telangiectasia resolution. Our results should assist the ophthalmologist in predicting treatment difficulties or poorer prognoses for Coats' patients with retinal cyst.

## Synopsis

123 children with Coats' who underwent wide-angle FA were retrospectively reviewed and results showed 18.70% (23/123) of the patients developed complications with retinal cyst and patients with retinal cyst presented with more clock-hours of telangiectasia on FA and needed more total treatments and longer time for telangiectasia resolution.

## Data Availability Statement

The raw data supporting the conclusions of this article will be made available by the authors, without undue reservation.

## Ethics Statement

The studies involving human participants were reviewed and approved by the Ethics Committee of Beijing Tongren Hospital. Written informed consent to participate in this study was provided by the participants' legal guardian/next of kin.

## Author Contributions

HL and SL: conceptualization and methodology. J-HL: data collection and writing original draft preparation. JM, LL, and GD: patient reviewing. YF: image preparation and editing. All authors contributed to the article and approved the submitted version.

## Funding

This study was supported by Beijing Tongren Hospital, Capital Medical University (TRZDYXZY201703).

## Conflict of Interest

The authors declare that the research was conducted in the absence of any commercial or financial relationships that could be construed as a potential conflict of interest.

## Publisher's Note

All claims expressed in this article are solely those of the authors and do not necessarily represent those of their affiliated organizations, or those of the publisher, the editors and the reviewers. Any product that may be evaluated in this article, or claim that may be made by its manufacturer, is not guaranteed or endorsed by the publisher.
